# Fourier transform infrared spectroscopy study of ligand photodissociation and migration in inducible nitric oxide synthase

**DOI:** 10.12688/f1000research.5836.2

**Published:** 2014-12-12

**Authors:** Michael Horn, Karin Nienhaus, Gerd Ulrich Nienhaus

**Affiliations:** 1 Karlsruhe Institute of Technology (KIT), Institute of Applied Physics, Karlsruhe, D-76131, Germany; 2 Department of Physics, University of Illinois at Urbana-Champaign, Urbana, IL, 61801, USA

**Keywords:** iNOS, migration, ligand, heme binding, FTIR

## Abstract

Inducible nitric oxide synthase (iNOS) is a homodimeric heme enzyme that catalyzes the formation of nitric oxide (NO) from dioxygen and L-arginine (L-Arg) in a two-step process. The produced NO can either diffuse out of the heme pocket into the surroundings or it can rebind to the heme iron and inhibit enzyme action. Here we have employed Fourier transform infrared (FTIR) photolysis difference spectroscopy at cryogenic temperatures, using the carbon monoxide (CO) and NO stretching bands as local probes of the active site of iNOS. Characteristic changes were observed in the spectra of the heme-bound ligands upon binding of the cofactors. Unlike photolyzed CO, which becomes trapped in well-defined orientations, as indicated by sharp photoproduct bands, photoproduct bands of NO photodissociated from the ferric heme iron were not visible, indicating that NO does not reside in the protein interior in a well-defined location or orientation. This may be favorable for NO release from the enzyme during catalysis because it reduces self-inhibition. Moreover, we used temperature derivative spectroscopy (TDS) with FTIR monitoring to explore the dynamics of NO and carbon monoxide (CO) inside iNOS after photodissociation at cryogenic temperatures. Only a single kinetic photoproduct state was revealed, but no secondary docking sites as in hemoglobins. Interestingly, we observed that intense illumination of six-coordinate ferrous iNOS _oxy_-NO ruptures the bond between the heme iron and the proximal thiolate to yield five-coordinate ferric iNOS _oxy_-NO, demonstrating the strong trans effect of the heme-bound NO.

## Introduction

**Figure 1.**
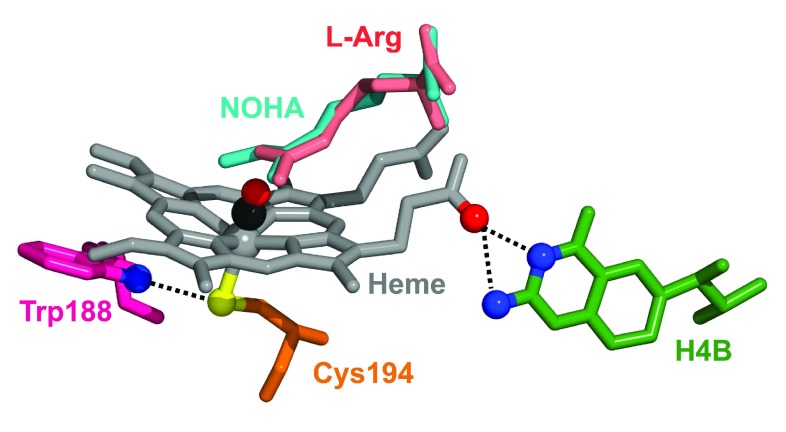
Schematic depiction of the iNOS _oxy_ active site (pdb codes 1NOD and 1DWV). The CO ligand was added for illustration.

Nitric oxide synthases (NOSs) are homodimeric heme enzymes that catalyze the oxidative degradation of L-arginine (L-Arg) to nitric oxide (NO) ^1, 2^. Three structurally similar NOS isoforms have been identified in endothelial cells (eNOS), neuronal tissues (nNOS) and in macrophages (iNOS) ^3^. Different from eNOS and nNOS, iNOS is not expressed in resting cells but induced upon inflammatory and immunologic stimulation. Each NOS protomer consists of an oxygenase and a reductase domain. In the catalytic oxygenase domain (NOS _oxy_), dioxygen (O _2_) binds to a central heme prosthetic group, anchored to the polypeptide chain via a proximal cysteine residue ( Figure 1). Its thiol sulfur atom accepts a hydrogen bond from an adjacent tryptophan residue. The substrate, L-Arg, is accommodated directly on top of the heme plane in the distal pocket; the cofactor tetrahydrobiopterin, H4B, binds along the side of the heme ^4– 7^. L-Arg and H4B are linked through an extended hydrogen bonding network mediated by one of the heme propionate groups. The reductase domain, NOS _red_, binds flavin mononucleotide (FMN), flavin adenine dinucleotide (FAD), and reduced nicotinamide adenine dinucleotide phosphate (NADPH). It provides the electrons for the catalytic reaction proceeding in the oxygenase domain. In a first step, L-Arg is converted to N-hydroxy L-Arg (NOHA). Subsequently, NOHA is decomposed into citrulline and nitric oxide (NO). Electron transfer is enabled by calmodulin binding in the interface between the two domains ^8^.

The NO molecule generated during enzymatic turnover can either coordinate directly to the heme iron or diffuse out of the protein into the environment. From there, it may again bind in a bimolecular process ^9^. Formation of the very stable ferrous NO complex results in self-inhibition of the enzyme. The probability of forming this product depends on the dissociation rate coefficient of NO from the ferric heme, the likelihood of autoreduction of the ferric NO-bound form to the ferrous derivative with its much stronger NO affinity, and the probability of oxidizing the ferrous NO-bound species to the ferric form plus nitrate by O _2_^10^. Deactivation of the enzyme may also occur via nitrosylation of the side chains of two cysteine residues coordinating a zinc ion in the dimer interface, which leads to irreversible dissociation into non-functional monomers ^11– 14^.

The iNOS isoform has been implicated in the pathogenesis of various diseases; so there is a growing interest in developing potent and highly selective inhibitors ^15, 16^. Their targeted design requires detailed insights into the interactions between ligand, substrate and the surrounding protein matrix. Therefore, we have investigated ligand and substrate binding in the iNOS oxygenase domain, iNOS _oxy_, by using Fourier transform infrared (FTIR) spectroscopy of the stretching vibrations of carbon monoxide (CO) and NO as ligands rather than the physiological ligand O _2_. They are of similar size as O _2_, which suggests that ligand dynamics within the protein may be comparable for all three ligands. CO and NO both have excellent properties as infrared (IR) spectroscopic probes ^17^. CO has proven to be an attractive heme ligand because the CO bond stretching vibration gives rise to strong mid-IR absorption bands that can be measured with exquisite sensitivity and precision ^17, 18^. The IR bands are fine-tuned by electrostatic interactions with the environment ^19– 21^; therefore, CO is frequently utilized as a local probe of protein structure and dynamics ^22^.

In the gas phase, CO absorbs at 2143 cm ^-1^ ^23^. When bound to the central iron of a heme cofactor, the CO stretching frequency, ν _CO_, which is typically in the 1900 – 2000 cm ^-1^ spectral range, is susceptible to changes in the iron-ligand bond and the local electric field due to the vibrational Stark effect ^24– 29^. There are two major contributions to the heme iron-CO bond, *i.e.*, σ-donation from a weakly antibonding 5σ MO of CO to the iron 4s and 3d _z_^2^ orbitals and π-backbonding from the iron 3d _z_ orbitals to the strongly antibonding CO 2π* orbital ^30^. A positive charge located near the CO oxygen attracts electron density, causing a decrease in σ-donation and an increase in backbonding. Consequently, the C–O bond order is reduced and ν _CO_ shifts to lower values ^19– 21^. A negative charge has the opposite effect.

After photodissociation of CO-bound heme protein samples, the stretching bands of unbound CO trapped inside a protein are found within the range from ~2080 to ~2160 cm ^-1^ ^18, 31^. The vibrational bands can reveal changes related to ligand relocation to other sites within the protein ^18, 29, 32, 33^, rotational motions of the ligand in these sites ^25, 34^ and protein conformational changes ^35^. Often, there are doublets of bands corresponding to opposite orientations of the CO at a particular transient docking site ^27, 29, 32, 36– 38^. The bond order and, therefore, ν _CO_ increases if the carbon atom interacts with a hydrogen bond donor, whereas an interaction with the ligand oxygen reduces both the bond strength and the stretching frequency ^29^.

Unlike CO, which only binds to a ferrous (Fe ^II^) heme iron, NO may coordinate to both the ferrous and the ferric (Fe ^III^) forms. So far, FTIR studies using NO have remained scarce because of its weaker intrinsic absorption. Furthermore, there is spectral overlap with the amide bands and ultrafast recombination of a major fraction of proteins even at very low temperatures. Therefore, only small photoproduct yields are obtained in experiments probing longer times such as FTIR, which renders experiments with ferrous NO technically challenging. Consequently, we have limited ourselves to NO binding to ferric heme in this work. For iNOS, this complex is of physiological relevance because the heme iron is in the ferric state after completion of the catalytic cycle.

Here, we have performed FTIR studies on iNOS at cryogenic temperatures, at which ligand rebinding is very slow. Thus, photoproducts induced by illumination are long-lived and can be conveniently studied by photolysis difference spectroscopy. Moreover, essentially all protein (and solvent) motions are frozen in ^39, 40^, so the ligands cannot escape to the solvent and can be observed within the protein matrix. We have combined FTIR with temperature-derivative spectroscopy (TDS) ^41– 43^, which allows us to disentangle photolysis-induced absorption changes caused by the different types of ligand dynamics.

## Materials and methods

### Protein expression

The iNOS _oxy_ domain, with its first 65 residues deleted (Δ65 iNOS _oxy_, referred to as iNOS _oxy_ in the following), was expressed essentially as described ^44^. Briefly, iNOS _oxy_ containing plasmids (pCWori) were transformed into competent *Escherichia coli* cells (strain BL21). The cells were plated on agar in the presence of 390 µM ampicillin (Carl Roth, Karlsruhe, Germany) and cultured overnight at 37°C. A single colony was added to 150 ml terrific broth (TB, Carl Roth) supplemented with ampicillin (390 µM) and agitated for 12 h at 37°C and 250 rpm. 10 ml of the overnight culture were added to 1.5 l TB, containing 390 µM ampicillin, and grown to an optical density of ~1 at 600 nm. Then, the temperature was lowered to 30°C and δ-aminolevulinic acid (44 µM, Sigma-Aldrich, St. Louis, MO, USA) and hemin (8 µM, Sigma-Aldrich) were added. iNOS expression was induced by adding isopropyl β-D-1-thiogalactopyranoside (IPTG, Carl Roth) to a final concentration of 100 µM. After 48 h (fresh ampicillin was added every 16 h), the cells were harvested by centrifugation for 20 min at 4°C and 2,000 rpm (swing-bucket rotor, 4–16 K, Sigma, Osterode, Germany). The cells were resuspended in lysis buffer (40 mM HEPES, 10% glycerol (vol.), 200 mM NaCl, pH 7.6, Carl Roth), mixed with 2 mg DNase (Sigma-Aldrich), and ruptured using a bead-beater (Biospec, Bartlesville, USA), filled with 0.1 mm (diameter) zirconia/silica beads (three treatments of 2 min each). The lysate was separated from the beads by a glass filter and loaded onto an immobilized-metal ion affinity column equilibrated with lysis buffer (Ni Sepharose 6 FastFlow, GE Healthcare). After washing with lysis buffer supplemented with increasing concentrations of imidazole (0, 10, 40 mM, Sigma-Aldrich), the protein was eluted with lysis buffer containing 160 mM imidazole. Appropriate fractions were pooled, dialyzed against water and concentrated by using Vivaspin Turbo 15 (cut-off 10 kDa) centrifugal concentrators (Sartorius, Göttingen, Germany). Finally, the protein was lyophilized and stored at -20°C.

### Sample preparation

To prepare CO-ligated iNOS _oxy_, 12 mg freeze-dried iNOS were slowly added to 40 µl cryosolvent (75%/25% glycerol/100 mM potassium phosphate buffer (v/v), pH 7.4, and, if so desired, supplemented with L-Arg and NOHA substrate (Sigma-Aldrich) or H4B cofactor (Sigma-Aldrich) to reach final concentrations of 200 mM and 100 mM, respectively) and stirred under 1 atm CO for 60 min. Subsequently, a two-fold molar excess of an anaerobically prepared sodium dithionite solution (Sigma-Aldrich) was added with a gas-tight Hamilton syringe, and the solution was stirred for another 10 min. To remove any undissolved protein, the solution was centrifuged for 10 min at 13,400 rpm (Minispin centrifuge, Eppendorf, Hamburg, Germany) before loading it into the sample cell. For an NO-ligated sample, ferric iNOS _oxy_ was dissolved in cryosolvent and stirred under an N _2_ atmosphere for 1 h. The gas phase above the sample was replaced repeatedly by N _2_ to efficiently remove O _2_. Finally, a few microliters of NO gas were added with a gas-tight syringe. NO ligation to the heme iron was confirmed by UV/vis absorption spectroscopy.

### Experimental setup

A few microliters of the sample solution were sandwiched between two CaF _2_ windows (diameter 25.4 mm) separated by a Mylar washer. The windows were mounted inside a block of oxygen-free high-conductivity copper. The copper block was attached to the cold-finger of a closed-cycle helium refrigerator (model F-50, Sumitomo, Tokyo, Japan). The sample temperature was measured with a silicon temperature sensor diode and regulated in the range 3 – 320 K by a digital temperature controller (model 336, Lake Shore Cryotronics, Westerville, OH). A continuous-wave, frequency-doubled Nd-YAG laser (Samba, Cobolt, Solna, Sweden), emitting up to 300 mW output power at 532 nm, was used to photolyze the sample. The laser beam was split and focused with lenses on the sample from both sides. Transmission spectra were recorded on a Vertex 80v FTIR spectrometer (Bruker, Karlsruhe, Germany) at a resolution of 2 cm ^–1^, using either an InSb detector (75 µm thick Mylar, 1,700 to 2,300 cm ^–1^) or an MCT detector (<5 µm thick Mylar, 1,100 to 2,300 cm ^–1^).

### FTIR photolysis difference spectroscopy

The infrared absorption of CO and NO can be studied selectively by using photolysis difference spectroscopy, which involves measurement of IR transmission spectra, *I*( *ν, T*), before and after photolysis. The difference absorbance of the two spectra, Δ *A*( *ν, T*) = log( *I*_dark_/ *I*_light_), contains only features that are due to photodissociation of the ligand from the heme iron. The missing absorption of the heme-bound ligands (A bands) after photolysis and the corresponding absorption of the photolyzed ligands (photoproduct bands) are displayed with negative and positive amplitudes, respectively. Peak positions and fractional occupancies were determined by fits with Gaussian band shapes; they are compiled in Table 1. In the following, we use the Gaussian band positions (frequencies) at 4 K as a subscript to ‘A’ (denoting the heme-bound state) to distinguish the absorbance bands and also to refer to a particular substate of the protein.

**Table 1.** Positions and fractional areas of the IR stretching bands of heme-bound and photodissociated CO and NO ligands in iNOS _oxy_ samples, determined at 4 K with estimated experimental errors of ± 0.5 cm ^-1^ and ± 3%, respectively.

	Heme-bound CO		Photolyzed CO (10 s @ 4 K)		Photolyzed CO (Slow cool)		Heme-bound NO		NO Photoproduct	
	cm ^-1^	%	cm ^-1^	%	cm ^-1^	%	cm ^-1^	%	cm ^-1^	%
**w/o substrate**	1921	9	2124	55	2124	85	1870	100	1814	23
	1945	40	2129	45	2132	15			1818	77
	1959	51								
**L-Arg**	1904	69	2120	28	2120	20	1829	56	1814	13
	1921	13	2131	28	2131	34	1847	16	1822	87
	1951	18	2144	32	2145	30	1870	28		
			2150	12	2150	16				
**NOHA**	1903	13	2122	75	2117	20	1851	15	1814	30
	1937	57	2133	19	2124	44	1870	85	1818	70
	1956	30	2145	6	2133	36				
**H4B**	1924	18	2124	75	2122	10	1872	82		
	1951	82	2133	25	2126	61	1890	18		
					2134	29				

Different illumination protocols were applied for photodissociation ^17^. Before starting a TDS experiment, the sample was illuminated for 10 s at 4 K to trap the photolyzed ligand close to the heme iron at the so-called primary docking site B. Alternatively, under ‘slow-cool’ illumination, the sample was cooled from 160 to 4 K at a rate 0.3 K/min under constant laser illumination to enable the photodissociated ligands to sample alternative docking sites that may not be accessible upon photolysis at 4 K. In both protocols, 300 mW laser power at 532 nm was used. To monitor the photodissociation kinetics, the samples were continuously illuminated for 15,000 s at reduced laser power (0.3 mW or 10 mW), and transmission spectra were recorded continuously. For comparison, the photolysis yield was scaled with respect to complete photodissociation with full laser power (300 mW).

### Temperature derivative spectroscopy (TDS)

TDS, an experimental protocol designed to study thermally activated rate processes involving enthalpy barrier distributions, has been described in great detail elsewhere ^41– 43^. Briefly, a non-equilibrium state is created in the sample at low temperature, *e.g.*, by photolysis with visible light. The integrated absorbance, *A*, of a spectral band taken at the lowest temperature represents the total photolyzed population, *N*. Subsequently, thermal relaxation of the sample back to equilibrium is recorded while the sample temperature is ramped up linearly over a few hours in the dark. One FTIR transmission spectrum is taken for every 1-K temperature increase. In the simplest analysis, we assume that any change in integrated absorbance is due to ligand rebinding and, therefore, proportional to a population change, Δ *N*, during acquisition of two successive spectra. TDS data are conveniently presented as two-dimensional contour plots, with solid lines indicating an absorbance increase and dashed lines a decrease. Contours are spaced logarithmically to emphasize small features.

## Results and discussion

### 1. FTIR spectroscopy of iNOS _oxy_ using CO as an internal probe

In the following, we present 4-K FTIR photolysis difference spectra of iNOS _oxy_-CO and briefly discuss the influence of substrate, substrate intermediate and cofactor on the CO stretching vibration and rebinding. For additional information, we refer to Jung *et al*. ^45^ and Li *et al.* ^46^.

**Figure 2.**
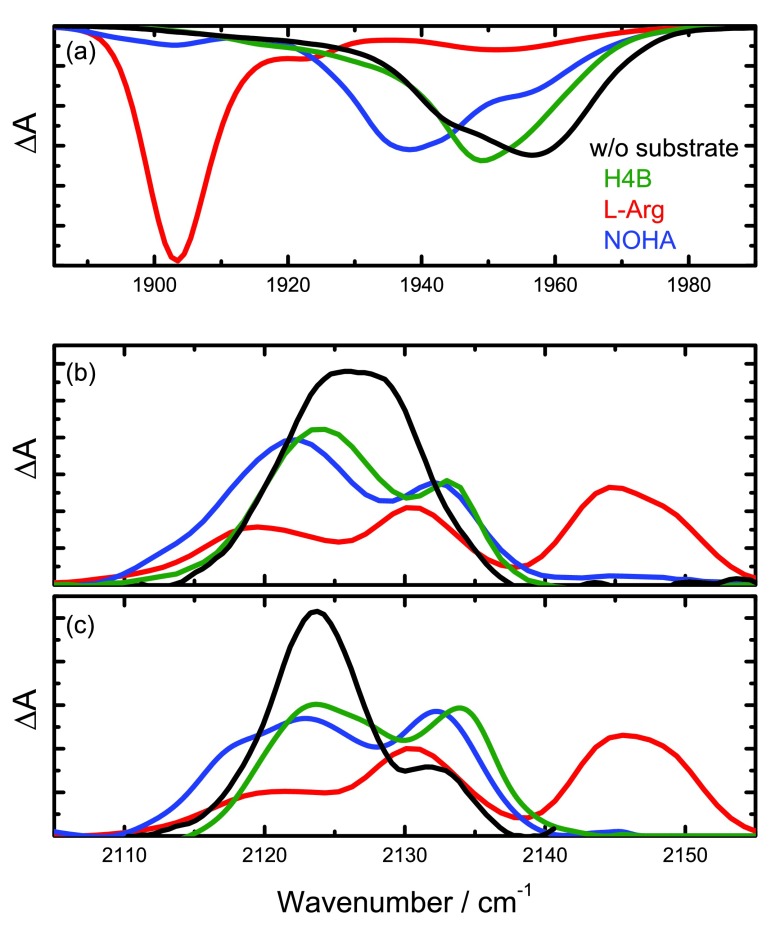
4-K photolysis difference spectra of iNOS _oxy_-CO. ( **a**) Absorption of the heme-bound CO. ( **b**) Photoproduct bands obtained after 10-s illumination at 4 K. ( **c**) Photoproduct bands obtained after constant illumination during slow cooling from 160 to 4 K.

***Photolysis difference spectra at 4 K.*** The 4-K absorption difference spectrum of iNOS _oxy_-CO displays two broad, extensively overlapping A bands at 1945 and 1959 cm ^–1^, indicative of two active site subconformations with significant intrinsic structural heterogeneity ( Figure 2a). Adding the H4B cofactor induces only small changes; the resulting spectrum can be described by a dominant A band centered on 1951 cm ^-1^ and a minor one at 1924 cm ^-1^ ( Figure 2a). As H4B binds along the side of the heme ^4^ and, thus, not in the immediate vicinity of the heme-bound CO, it is not expected to modify ν _CO_ to any significant extent. In contrast, the presence of L-Arg shifts the main A band of iNOS _oxy_-CO/L-Arg to 1904 cm ^-1^; smaller features are located at 1921 and 1951 cm ^-1^ ( Figure 2a). The pronounced red-shift of A _1904_ arises from the electron-withdrawing effect of the terminal, positively charged NH _2_^+^ moiety of the L-Arg side chain close to the bound CO ^4^. The position of the A _1921_ band is indicative of an electrostatic interaction of the CO dipole with a less pronounced positive partial charge, most likely the neutral terminal amino group of the L-Arg side chain.

If the reaction intermediate NOHA is present, three A bands at 1903, 1937 and 1956 cm ^-1^ are discernable ( Figure 2a). The crystal structure shows that NOHA binds in the same orientation in the active site as L-Arg, with the side chain pointing towards the heme iron ^47^. Therefore, we suggest that, in those iNOS _oxy_ molecules absorbing within the A _1937_ band, a hydrogen bonding interaction exists between the CO ligand and the hydroxyl group of the NOHA side chain. A _1903_ is most likely associated with iNOS _oxy_ molecules, in which the terminal amine of the NOHA side chain is protonated (p *K* = 8.1 ^48^) and points towards the heme-bound CO. The protonated NOHA has been suggested to be the catalytically active substrate intermediate ^49, 50^.

The absorption spectra of photolyzed CO are plotted in Figures 2b (brief illumination at 4 K) and 2c (slow-cool illumination); peak positions and relative areas are included in Table 1. For comparison, the integrated absorption in each spectral region was scaled to the same area. We note that the ratio of the integrated areas of the A and photoproduct bands is ~20 ^18^.

All photoproduct spectra obtained after 10-s illumination at 4 K have absorption bands in the 2120 – 2130 cm ^-1^ spectral range ( Figure 2b). The spectrum of iNOS _oxy_-CO is composed of two stretching bands at 2124 and 2129 cm ^-1^. With H4B, photoproduct bands appear at 2124 and 2133 cm ^-1^, indicating that the cofactor has an effect on ν _CO_ of the unbound CO. In the presence of L-Arg, the absorption bands are centered on 2120 and 2131 cm ^-1^, and there are two additional bands at 2144 and 2150 cm ^-1^. Their higher stretching frequencies suggest formation of a hydrogen bond between the ligand carbon and the terminal amine group of L-Arg ^29^. Upon NOHA binding, the photoproduct bands are centered on 2122 and 2133 cm ^-1^. The minor absorption at 2145 cm ^-1^ can be associated with CO ligands photolyzed from iNOS _oxy_/NOHA trapped in its A _1903_ conformation.

The photoproduct spectra obtained after slow-cool illumination ( Figure 2c) are similar to the ones recorded after 10-s illumination ( Figure 2b), suggesting that it is not possible to populate additional docking sites to any significant extent. The greatest difference is seen for iNOS _oxy_-CO. Its photoproduct spectrum shows two well separated bands at 2124 and 2134 cm ^-1^ rather than the non-separated doublet seen in Figure 2b. We also note that there is an additional shoulder at 2117 cm ^-1^ for iNOS _oxy_-CO/NOHA.

**Figure 3.**
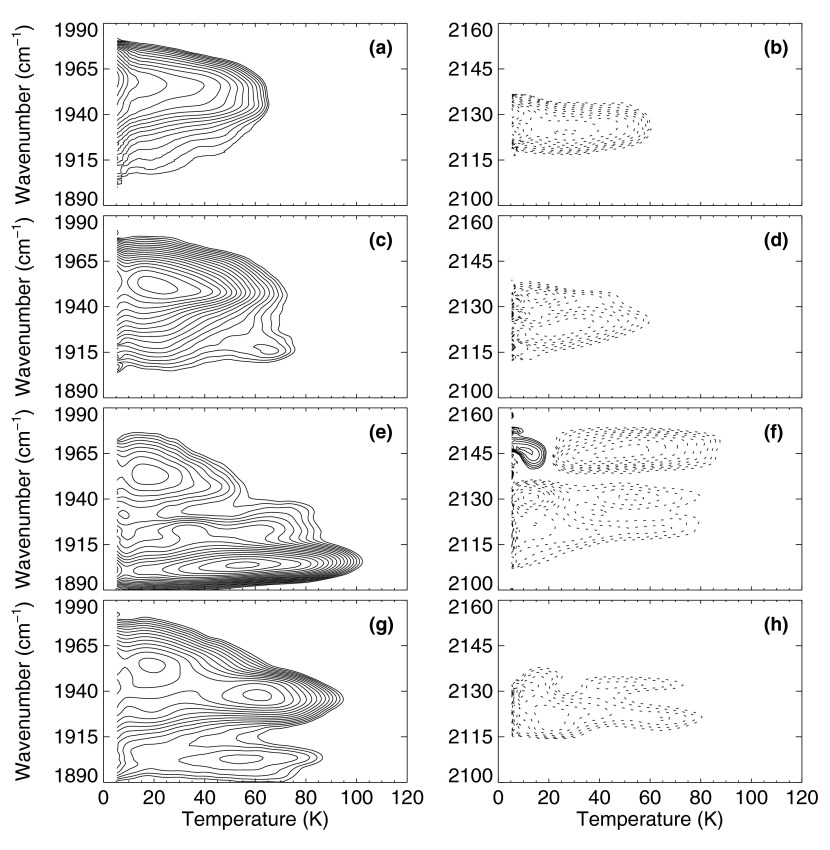
TDS contour maps of iNOS _oxy_-CO, obtained after 10-s illumination at 4 K. Left column: Absorption changes in the bands of heme-bound CO. Right column: Absorption changes in the photoproduct bands. Contours are spaced logarithmically; solid and dotted lines represent increasing and decreasing absorption, respectively. iNOS _oxy_-CO ( **a**, **b**) w/o substrate; ( **c**, **d**) with H4B; ( **e**, **f**) with L-Arg; ( **g**, **h**) with NOHA.

***CO rebinding in iNOS _oxy_.*** To obtain more information on the photoproduct states, TDS measurements were started at 4 K immediately after illumination. Figure 3 displays the contour maps obtained after 10-s illumination at 4 K, with the absorption changes in the A bands and the photoproduct bands in the left and right columns, respectively.

All iNOS _oxy_-CO samples display single-step CO rebinding. This observation indicates that there is only a single kinetic state of the photolyzed protein-ligand complex, and the presence of sharp photoproduct bands indicates that the photolyzed ligands are trapped in transient docking sites with well-defined orientations. In substrate-free iNOS _oxy_-CO, recombination is maximal at 4 K, indicating that there is substantial rebinding already at this low temperature. Rebinding extends up to ~70 K ( Figure 3a, b). Rebinding in the dominant A _1951_ substate of iNOS _oxy_-CO/H4B peaks at 20 K; as in iNOS _oxy_-CO, the process extends to 70 K. Only the minor A _1924_ subpopulation shows a focused rebinding peak at ~60 K ( Figure 3c). The photoproduct map does not yield additional information ( Figure 3d). Binding of either L-Arg or NOHA in the active site shifts CO rebinding to higher temperatures, suggesting that the hydrogen bonding interaction stabilizes the ligands at the transient docking site against rebinding ( Figures 3e and 3g). Maximal rebinding in iNOS _oxy_/NOHA, *i.e.,* in A _1903_ and A _1937_, occurs at 50 – 60 K ( Figure 3g). The corresponding photoproduct bands are centered on 2122 and 2133 cm ^-1^ ( Figure 3h). The contours at 1950 – 1960 cm ^-1^ ( Figure 3g) represent rebinding in the NOHA-free A _1956_ substate. With L-Arg anchored in the active site, CO ligands return to the heme iron also at ~50 – 60 K ( Figure 3e). The corresponding photoproduct map shows a concomitant loss of the photoproduct bands at 2150, 2144, 2131 and 2120 cm ^-1^, associating these bands with CO molecules trapped in the vicinity of the substrate ( Figure 3f). A population transfer between photoproduct states due to CO rotation ^32, 51, 52^ is apparent from the mirror-imaged dashed and solid contours at 2131 and 2144 cm ^-1^ at 12 K. The solid contours at ~2144 cm ^-1^ in Figure 3f indicate a growth of this photoproduct population during the TDS measurement. Because data are taken in the dark, this can only occur via an exchange of photoproduct population from one band to another because of dynamics. Here, photoproduct population transfers from 2131 cm ^-1^ to ~2144 cm ^-1^, and the underlying process is most likely a rotation of the CO by 180° so as to attain thermal equilibrium between the two states corresponding to opposite orientations of the CO ^17^.

**Figure 4.**
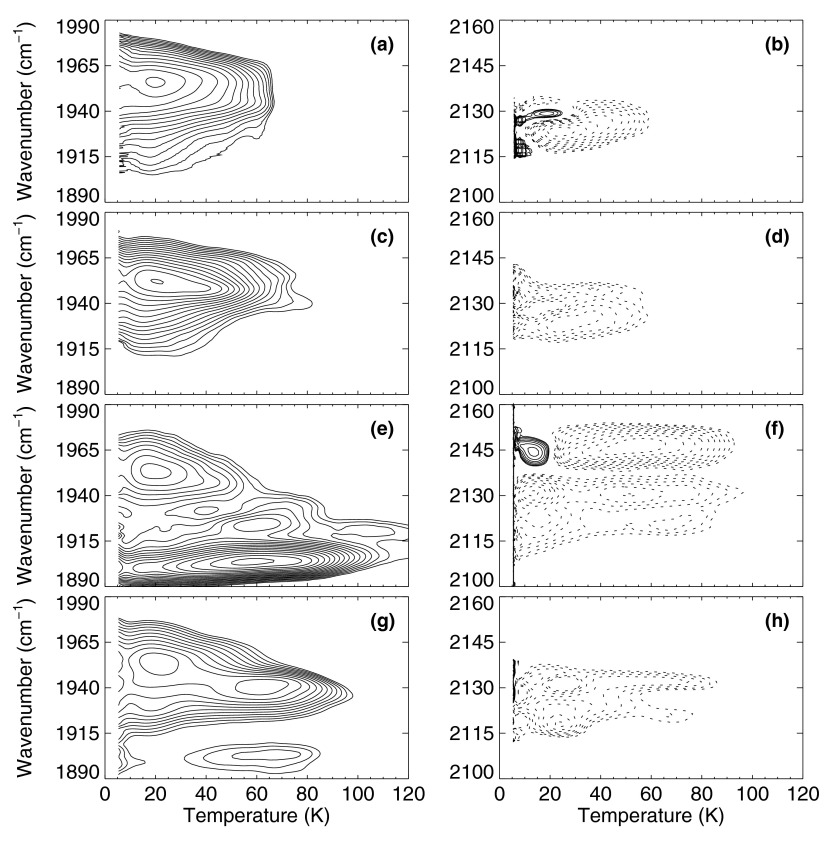
TDS contour maps of iNOS _oxy_-CO, obtained after constant illumination during slow cooling from 160 to 4 K. Left column: Absorption changes in the bands of heme-bound CO. Right column: Absorption changes in the photoproduct bands. Contours are spaced logarithmically; solid and dotted lines represent increasing and decreasing absorption, respectively. iNOS _oxy_-CO ( **a**, **b**) w/o substrate; ( **c**, **d**) with H4B; ( **e**, **f**) with L-Arg; ( **g**, **h**) with NOHA.

The TDS maps after slow-cool illumination ( Figure 4) show only marginal differences to the ones obtained after brief 4-K illumination ( Figure 3), which confirms that the photodissociated CO ligands populate only a single kinetic state. Notably, after slow-cool illumination, rebinding generally occurs at slightly higher temperatures than after brief 4-K illumination. The observed slowing may be attributed to small structural changes near the active site, causing an increase of the ligand binding barrier. A similar effect was also visible in MbCO upon extended illumination below 40 K ^42^ as well as in NO- and CO-ligated nitrophorin 4 ^35^.

In a typical globin protein involved in ligand transport or storage, the primary ligand docking site B is indispensable because it ensures efficient ligand binding to and release from the heme iron ^53^. Incoming ligands are ‘caught’ in site B before the actual bond formation process occurs ^32, 54^. Upon thermal dissociation from the heme iron, ligands can remain unbound in site B for some time, which increases their probability to escape from the protein. Without this site, they would immediately recombine with the heme iron, as is, *e.g*., observed for NO-transporting nitrophorin ^35^ and modified cytochrome c ^55^.

The catalytic reaction of iNOS requires sequential binding of two O _2_ molecules and efficient release of the NO product. Therefore, the B site is likely to have dual functionality. On the one hand, it allows efficient O _2_ binding to the heme iron. On the other hand, it ensures efficient release of the generated NO. Using CO as a ligand, we have shown that the B site is readily accessible for ligands photodissociated from the heme iron, both in the presence and absence of L-Arg or NOHA. The substrates stabilize the CO ligand at the transient site via hydrogen bonding. This stabilizing effect is also seen for the minor A _1924_ subpopulation of iNOS _oxy_/H4B. Presumably, a small fraction of H4B molecules are positioned such that they can form a direct hydrogen bond.

### 2. FTIR spectroscopy of iNOS _oxy_ using NO as an internal probe

The NO stretching absorption is also very suitable as a local probe of the active site structure and of ligand movements within a protein ^17^. Despite their similar sizes, the ligands may show different dynamics inside the protein ^56^. For example, in myoglobin (Mb), a transient docking site on the proximal side of the heme is readily populated by CO but not at all by NO ^56^. Such subtle differences could be relevant for the inhibitory effects of NO. Therefore, we have analyzed NO binding in ferric iNOS _oxy_ using FTIR-TDS at cryogenic temperatures.

**Figure 5.**
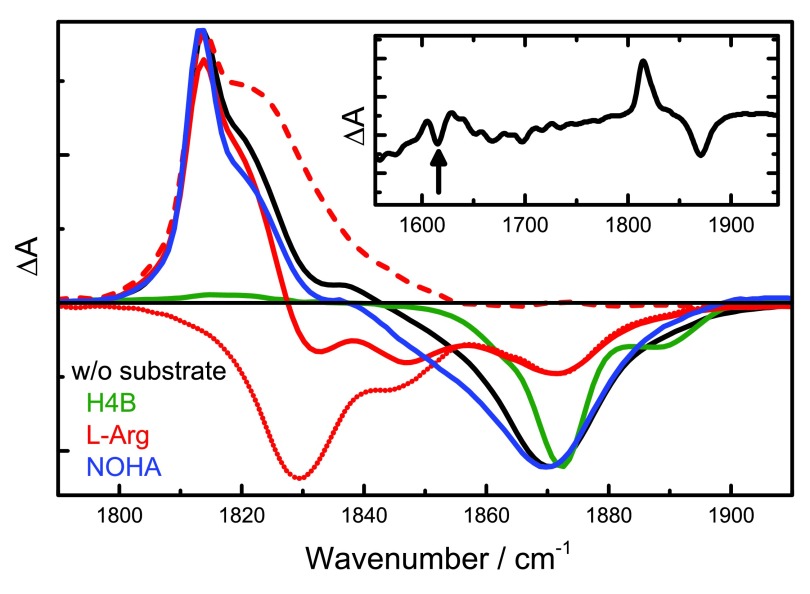
4-K photolysis difference spectra of iNOS _oxy_-NO. The stretching bands of heme-bound NO (NO after laser illumination at 300 mW) are plotted with negative (positive) amplitude. A bands of the NOHA spectrum were scaled independently of photoproducts (factor 2.07) to match the A bands of the spectrum without substrate. Dotted line: 4-K photolysis difference spectrum of iNOS _oxy_-NO/L-Arg, obtained upon illumination at 0.3 mW. Dashed line: iNOS _oxy_-NO/L-Arg photoproduct spectrum (obtained by calculating the difference between the two iNOS _oxy_-NO/L-Arg spectra). Inset: extended 4-K photolysis difference spectrum of iNOS _oxy_-NO.

***Photolysis difference spectra at 4 K.*** Figure 5 displays 4-K photolysis difference spectra of various ferric iNOS _oxy_-NO preparations. Most spectra show an A band at 1870 cm ^-1^ associated with NO bound in an active site without bound cofactor or substrate ( Table 1). In the spectrum of iNOS _oxy_-NO, A _1870_ is rather broad, suggesting significant conformational heterogeneity at the active site. The spectrum of iNOS _oxy_-NO/NOHA is very similar, dominated by the broad A _1870_ band; the only clear change from iNOS _oxy_-NO is a shoulder at 1851 cm ^-1^. This comparison suggests that NOHA is bound only in a small subfraction reflected by the shoulder. In iNOS _oxy_-NO/L-Arg, A _1847_ and A _1829_ report the binding of L-Arg. A _1870_ is still present due to incomplete saturation with substrate ( Figure 5). Interestingly, Rousseau *et al*. ^2^ could not identify any changes of ν _Fe-N_ in their resonance Raman spectra upon binding of L-Arg and even hypothesized that L-Arg does not bind to ferric iNOS _oxy_-NO. With H4B anchored next to the heme, the A band is shifted to 1872 cm ^-1^, and another absorption band emerges at 1890 cm ^-1^.

Most of the observed spectral shifts can again be explained by backbonding ^57^ because the ferric NO-ligated ground state, which is best described as Fe ^II^NO ^+^, is isoelectronic to Fe ^II^CO ^58^. The heme-bound NO absorbs at 1870 cm ^-1^. L-Arg shifts ν _NO_ to lower frequencies; the A _1829_ and A _1847_ bands indicate an interaction between the NO and the positively charged and neutral terminal amino groups of the L-Arg side chain. As already observed for CO, the effect of NOHA is less pronounced; its presence is visible *via* a shift of the A band to 1851 cm ^-1^. Interestingly, the NO stretching absorption is also affected by H4B. The band shifts slightly and, in addition, it becomes rather narrow, which is indicative of a more homogeneous active site environment or restricted dynamics of the heme-bound NO due to the bound H4B ^35, 59^. In 2005, Rousseau *et al*. ^2^ reported that, upon H4B binding, a Raman band emerges that was assigned to the Fe-N-O bending mode, δ _Fe-N-O_, of the ferric adduct, indicating a more homogeneous bending of the bent NO. In thiolate-ligated Fe ^III^NO adducts, NO is typically bound at an angle of 160° ^60– 66^, and H4B binding next to the heme is not expected to modify this angle due to steric interactions. It may, however, restrict its librational dynamics around this angle, possibly because of the increased heme distortion caused by H4B ^67, 68^. The additional band at 1890 cm ^-1^ may indicate partial occupancy of a water molecule in the active site ^62^.

The photoproduct bands, displayed in Figure 5 with positive amplitudes, are in the 1810 – 1830 cm ^-1^ spectral range and, thus, red-shifted by only ~50 cm ^-1^ from those of the heme-bound NO ( Table 1). For iNOS _oxy_-NO/L-Arg, the photoproduct and A bands even overlap. Their decomposition (details are discussed below) yields a narrow photoproduct band at 1814 cm ^-1^ and a broad feature at 1822 cm ^-1^. iNOS _oxy_-NO and iNOS _oxy_-NO/NOHA show two photoproduct bands at 1814 and 1818 cm ^-1^. Interestingly, these bands are about as strong as the A bands, which strongly suggests that they do not represent unbound NO trapped in a transient docking site but rather heme-bound NO with restricted librational freedom.

In contrast to all other samples, the iNOS _oxy_-NO/H4B photoproduct spectrum reveals only a very weak feature at ~1818 cm ^-1^. This finding may be explained by a photolyzed NO that cannot be trapped in well-defined orientations. As a result, the stretching absorption becomes extremely broad and hardly distinguishable from the background. A similar effect was observed for NO in the primary photoproduct site B of ferric Mb ^56^.

**Figure 6.**
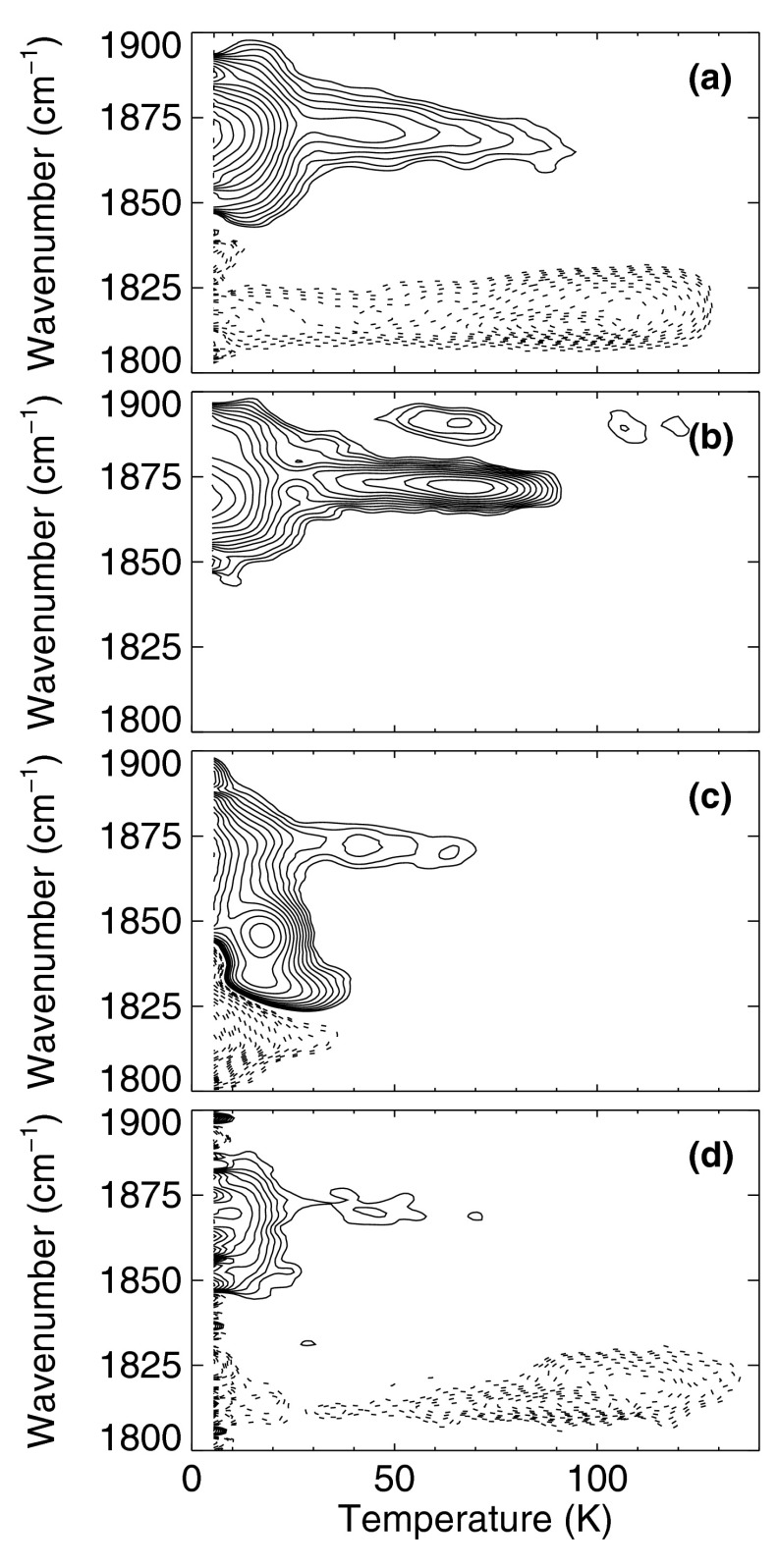
TDS contour maps of iNOS _oxy_-NO, obtained after 30-min illumination at 4 K. iNOS _oxy_-NO ( **a**) w/o substrate; ( **b**) with H4B; ( **c**) with L-Arg; ( **d**) with NOHA. Contours are spaced logarithmically; solid and dotted lines represent increasing and decreasing absorption, respectively.

***NO rebinding in ferric iNOS _oxy_.*** To gain additional information on the peculiar, strongly absorbing NO photoproduct bands, TDS experiments were started immediately after illuminating NO-ligated samples at 4 K. Figure 6 displays the absorption changes in the A bands and in the photoproduct bands with solid and dotted lines, respectively. The contour maps obtained after slow cool illumination (not shown) are essentially identical, as for the CO-ligated samples.

In iNOS _oxy_-NO, NO rebinding in A _1870_ starts already at the lowest temperatures ( Figure 6a) and extends to ~90 K. The decay of the photoproduct, however, occurs predominantly between 80 and 120 K, indicating that these bands cannot be associated with NO ligands photolyzed from the ferric heme iron, as reported by the A _1870_ band. Apparently, laser illumination produces a photoproduct band from another NO species in the sample. The TDS map of iNOS _oxy_-NO/NOHA ( Figure 6d) shows essentially the same features. It is likewise evident that NO rebinding is complete below 80 K, whereas the strange photoproduct feature disappears in the temperature range 80 – 120 K. In iNOS _oxy_-NO/H4B ( Figure 6b), NO rebinding at the ferric iron also starts at 4 K. In a subpopulation, recombination peaks at ~65 K; absorption changes of photoproducts are too small to be detected. NO rebinding in the L-Arg-bound A _1829_ and A _1847_ substates occurs mainly below 30 K, concomitantly with the decay of the photoproduct ( Figure 6c). The apparent maximum in the contours at 15 K and ~1850 cm ^-1^ is artificial and results from the superposition of the A bands and the photoproduct bands (compare Figure 5). Recombination in the substrate-free A _1870_ fraction of the sample is maximal at 4 K and extends out to ~70 K, consistent with the data shown in Figure 6a.

In summary, rebinding of NO to the ferric heme of iNOS _oxy_ is a one-step process. The corresponding photoproduct bands, *i.e*., the absorption bands of NO photodissociated from the ferric heme, were not identifiable. Presumably, NO is bound only weakly within the protein, without any well-defined orientation and without any additional stabilization via hydrogen bonding interactions to the substrate or the cofactor. As a consequence, the NO has a broad stretching absorption that cannot be distinguished from the background. Note that, if the photoproduct bands were masked by the strong bands at ~1820 cm ^-1^, they should have become visible in the spectrum of iNOS _oxy_-NO/H4B ( Figure 5).

***Identification of the iNOS _oxy_-NO photoproduct.*** The TDS data in Figure 5 clearly prove that the strong absorption bands at ~1820 cm ^-1^ are not generated by photodissociation of NO bound to ferric heme, absorbing at ~1870 cm ^-1^. To identify the corresponding pre-illumination states, we screened the 4-K FTIR photolysis difference spectrum of iNOS _oxy_-NO from 1,100 to 2,300 cm ^-1^ and detected a band at 1616 cm ^-1^, which we tentatively associate with a six-coordinate (6C) ferrous NO adduct ( Figure 5, inset). This assignment is supported by the ν _NO_ of 1591 cm ^-1^ reported for 6C ferrous P450 _cam_-NO ^69^. Praneeth *et al*. ^70^ also computed frequencies in this range, ν _NO_ = 1617 cm ^-1^ and ν _NO_ < 1600 cm ^-1^ for thiophenolate- and alkylthiolate-heme complexes, respectively, using density functional theory calculations on ferrous, thiolate-coordinated porphyrin model systems.

The admixture of a ferrous NO species in our samples does not come as a surprise. Ferric iNOS _oxy_-NO is unstable and spontaneously converts to a ferrous 6C NO-ligated species. This conversion may take place during loading and cooling of an FTIR sample, which typically takes ~2 h. This species may subsequently evolve further to a five-coordinate (5C) complex by dissociation of the thiolate ligand on time scales of minutes to hours, depending on the iNOS _oxy_ oligomerization state ^67, 71– 73^. Here, we can safely exclude formation of significant amounts of 5C ferrous iNOS _oxy_-NO because we have not observed the characteristic IR bands of this species at ~1670 cm ^-1^ ^53^.

**Figure 7.**
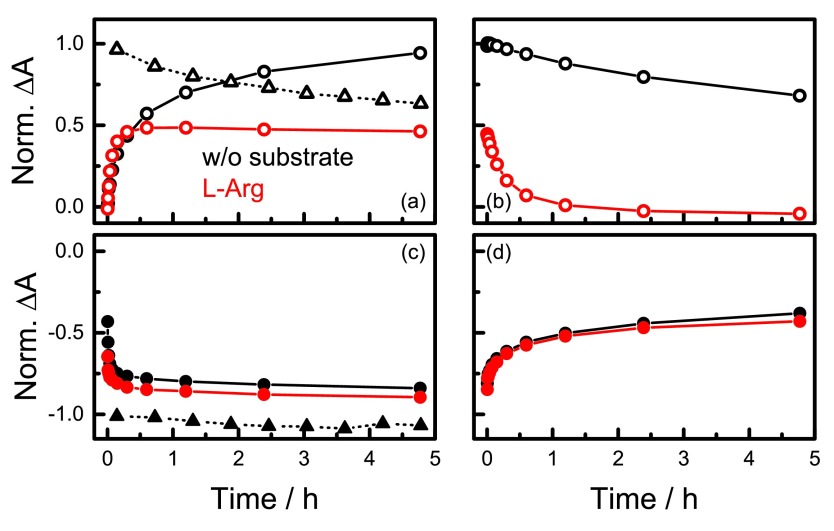
Temporal development of the integrated absorbance of the bands of 5C ferric iNOS _oxy_-NO (open symbols) and 6C ferric iNOS _oxy_-NO (filled symbols) ( **a**, **c**) during constant illumination at 4 K (circles: 10 mW, 532 nm; triangles: 300 mW, 532 nm) and ( **b**, **d**) after the laser was switched off. Black: iNOS _oxy_-NO; red: iNOS _oxy_-NO/L-Arg.

NO photodissociation from the 6C adduct is not expected to generate NO photoproduct bands that are of similar strength as the original A _1616_ band. Therefore, there must be yet another species responsible for the strong absorption at ~1820 cm ^-1^. Perhaps, light-induced breakage of the iron-sulfur rather than the iron-NO bond could lead to an alternative photoproduct, considering the strong *trans* effect exerted by the NO in 6C ferrous heme NO adducts ^66^. Indeed, Ibrahim *et al*. ^74^ had noticed earlier that laser light passing through solution samples of 6C ferrous model porphyrins adducts during resonance Raman measurements was sufficient to photodissociate the axial thiolate base *trans* to the NO ^75^. This effect could be suppressed by lowering the temperature to 77 K and reducing the laser power. Accordingly, we have illuminated the iNOS _oxy_-NO/L-Arg sample at low laser intensity (0.3 mW at 532 nm). This power was still sufficient to photodissociate the NO from the 6C ferric heme adduct (dotted line in Figure 5), photoproduct bands at ~1820 cm ^-1^, however, did not emerge, confirming that the photoproduct was not formed. Therefore, we propose that illumination of 6C ferrous iNOS _oxy_-NO with sufficient laser power leads to rupture of the bond between the iron and the proximal Cys194 thiolate, leaving behind a 5C iNOS _oxy_-NO. Because the NO is still bound to the heme iron, the intensity of the IR bands at ~1820 cm ^-1^ is comparable to that of other A bands ^25, 34^. The NO stretching frequency of the 5C adduct indicates that the ligand is coordinated to a ferric iron, so that the Cys194 sulfur is negatively charged after photodissociation. Similar NO stretching frequencies were reported for an isolated 5C ferric heme nitrosyl complex (ν _NO_ = 1842 cm ^-1^ ^76^) and for NO-ligated porphyrins with phenyl (ν _NO_ = 1825 cm ^-1^) and pentafluorophenyl (ν _NO_ = 1859 cm ^-1^) substituents on the four *meso* positions ^77^. If the laser power is sufficiently high (300 mW), it is even possible to photodissociate the NO from the 5C ferric iNOS _oxy_-NO, leaving behind a four-coordinate, ‘naked’ heme as a ‘secondary photoproduct’ ( Figure 7a).

L-Arg binding in the active site lowers the yield of ferric 5C iNOS _oxy_-NO upon laser illumination ( Figures 7a and c) and favors reformation of the iron-sulfur bond as soon as the laser is switched off ( Figures 7b and d). This effect may result from the competition between the NO ligand and the thiolate for σ charge donation to the heme iron; the higher the donation, the stronger the bond to the donor and the weaker the bond to the opposing heme ligand. The σ donor strength of the thiolate is altered by hydrogen bonding interactions to the sulfur atom ^66^. Using sulfur K-edge x-ray absorption spectroscopy and density functional theory calculations, Dey *et al*. ^78^ showed that each hydrogen bond reduces the electron-donating power of the thiolate sulfur. The NO electron donor ability and, therefore, its repulsive *trans* effect can be reduced by interactions that draw electron density away from the NO ^79, 80^, here by the hydrogen bonding interaction with L-Arg, so that the axial iron-sulfur bond is stabilized.

We also note that 6C ferrous iNOS _oxy_-NO is not stable in the presence of H4B but spontaneously oxidizes to the ferric form ^46^. Consequently, the yield of the 5C adduct is negligible, as is indicated by the low intensity of the absorption bands ( Figure 5).

***Ferric 5C iNOS _oxy_-NO.*** In view of the competition between the NO ligand and the thiolate for σ charge donation to the heme iron, one should expect ν _NO_ of the 5C photoproduct lacking the thiolate ligand to be blue-shifted with respect to ν _NO_ of the 6C adduct because the repulsive *trans* effect of the thiolate has been removed. Experimentally, however, the opposite behavior is observed ( Figure 5). To resolve this apparent discrepancy, one has to consider that the 5C ferric form originates from a 6C ferrous species, in which the NO is typically bound at an angle of ~140°. In the corresponding 6C ferric derivatives, the Fe – N – O angle is normally ~160°. At cryogenic temperatures, the dynamics of the protein matrix is completely arrested ^39, 40^.

Consequently, the NO is held in the strongly bent (lower angle) orientation of the 6C ferrous form. Based on DFT calculations, Linder *et al*. ^81^ reported that reducing the angle from 160° to 150° shifts ν _NO_ in 5C model porphyrins from 1897 to 1857 cm ^-1^. Therefore, we suggest that the low ν _NO_ of the 5C form is caused by NO binding at a small angle. We note that the similar ν _NO_ in 5C and 6C ferric iNOS _oxy_-NO/L-Arg implies that the bound substrate controls the angle at which the NO binds. Apparently, steric constraints override the bending induced by the trans effects.

Finally, we point out that, in contrast to the photo-induced 6C ferric → 5C ferric transition observed in the FTIR experiments at cryogenic temperatures, the spontaneous conversion of the 6C ferric NO-bound iNOS _oxy_ derivative at physiological temperatures involves two NO molecules and yields a 5C ferrous species ^71, 72, 82^. After binding the first NO, the ferric 6C iNOS _oxy_-NO reacts with a second ligand to yield 6C ferrous iNOS _oxy_-NO. This complex immediately converts to the 5C form and a nitrosonium ion (NO ^+^). The ion may diffuse towards the zinc binding site and nitrosylate one of the Cys residues involved in coordinating the zinc.

Fourier transform infrared photolysis difference spectra of CO- and NO-ligated inducible nitric oxide synthase Detailed information on the dataset can be found in the text file “Raw data legend”.

## Conclusions

FTIR spectroscopy at cryogenic temperatures, especially in combination with sophisticated illumination and data acquisition temperature protocols, provides quantitative data on protein-ligand interactions. Our FTIR-TDS studies on iNOS _oxy_ have shown that CO and NO rebinding involve only a single transient state in iNOS _oxy_. The CO is stabilized in well-defined orientations at the docking site by hydrogen bonding interactions and, therefore, gives rise to rather narrow photoproduct bands. In contrast, photoproduct bands associated with the photolyzed NO cannot be resolved. The NO appears to be trapped in less specific orientations, which may favor the release of this ligand. Under physiological conditions, release of the generated NO from the protein is facilitated.

Upon illumination of 6C ferrous iNOS _oxy_-NO at cryogenic temperatures, a 5C ferric NO adduct was identified, providing direct evidence for light-induced breakage of the iron-thiolate bond. Future studies along these lines are likely to contribute to a better understanding of functional processes in which the NO ligand is involved.

## Data availability

*F1000Research*: Dataset 1. Fourier transform infrared photolysis difference spectra of CO- and NO-ligated inducible nitric oxide synthase, 10.5256/f1000research.5836.d39481 ^83^

## References

[1] Li HY Poulos TL: Structure-function studies on nitric oxide synthases. *J Inorg Biochem.* 2005;99(1):293–305. 10.1016/j.jinorgbio.2004.10.016 15598508

[2] Rousseau DL Li D Couture M: Ligand-protein interactions in nitric oxide synthase. *J Inorg Biochem.* 2005;99(1):306–323. 10.1016/j.jinorgbio.2004.11.007 15598509

[3] Stuehr DJ: Mammalian nitric oxide synthases. *Biochim Biophys Acta.* 1999;1411(2–3):217–230. 10.1016/S0005-2728(99)00016-X 10320659

[4] Crane BR Arvai AS Ghosh DK: Structure of nitric oxide synthase oxygenase dimer with pterin and substrate. *Science.* 1998;279(5359):2121–2126. 10.1126/science.279.5359.2121 9516116

[5] Li H Raman CS Glaser CB: Crystal structures of zinc-free and -bound heme domain of human inducible nitric-oxide synthase. Implications for dimer stability and comparison with endothelial nitric-oxide synthase. *J Biol Chem.* 1999;274(30):21276–21284. 10.1074/jbc.274.30.21276 10409685

[6] Raman CS Li H Martasek P: Crystal structure of nitric oxide synthase heme domains. *J Inorg Biochem.* 1999;74:44–44. 10.1016/s0162-0134(00)00099-4 11051558

[7] Fischmann TO Hruza A Niu XD: Structural characterization of nitric oxide synthase isoforms reveals striking active-site conservation. *Nat Struct Biol.* 1999;6(3):233–242. 10.1038/6675 10074942

[8] Abu-Soud HM Yoho LL Stuehr DJ: Calmodulin controls neuronal nitric-oxide synthase by a dual mechanism. Activation of intra- and interdomain electron transfer. *J Biol Chem.* 1994;269(51):32047–32050. 7528206

[9] Abu-Soud HM Ichimori K Nakazawa H: Regulation of inducible nitric oxide synthase by self-generated NO. *Biochemistry.* 2001;40(2):6876–6881. 10.1021/bi010066m 11389602

[10] Santolini J Adak S Curran CM: A kinetic simulation model that describes catalysis and regulation in nitric-oxide synthase. *J Biol Chem.* 2001;276(2):1233–1243. 10.1074/jbc.M006858200 11038356

[11] Mitchell DA Erwin PA Michel T: S-Nitrosation and regulation of inducible nitric oxide synthase. *Biochemistry.* 2005;44(12):4636–4647. 10.1021/bi0474463 15779890

[12] Smith BC Fernhoff NB Marletta MA: Mechanism and kinetics of inducible nitric oxide synthase auto-S-nitrosation and inactivation. *Biochemistry.* 2012;51(5):1028–1040. 10.1021/bi201818c 22242685 PMC3277664

[13] Rosenfeld RJ Bonaventura J Szymczyna BR: Nitric-oxide synthase forms N-NO-pterin and S-NO-cys: implications for activity, allostery, and regulation. *J Biol Chem.* 2010;285(41):31581–31589. 10.1074/jbc.M109.072496 20659888 PMC2951232

[14] Ravi K Brennan LA Levic S: S-nitrosylation of endothelial nitric oxide synthase is associated with monomerization and decreased enzyme activity. *Proc Natl Acad Sci U S A.* 2004;101(8):2619–2624. 10.1073/pnas.0300464101 14983058 PMC356999

[15] Crane BR Arvai AS Gachhui R: The structure of nitric oxide synthase oxygenase domain and inhibitor complexes. *Science.* 1997;278(5337):425–431. 10.1126/science.278.5337.425 9334294

[16] Garcin ED Arvai AS Rosenfeld RJ: Anchored plasticity opens doors for selective inhibitor design in nitric oxide synthase. *Nat Chem Biol.* 2008;4(11):700–707. 10.1038/nchembio.115 18849972 PMC2868503

[17] Nienhaus K Nienhaus GU: Ligand dynamics in heme proteins observed by Fourier transform infrared spectroscopy at cryogenic temperatures. *Methods Enzymol.* 2008;437:347–378. 10.1016/S0076-6879(07)37018-3 18433637

[18] Nienhaus K Nienhaus GU: Ligand dynamics in heme proteins observed by Fourier transform infrared-temperature derivative spectroscopy. *Biochim Biophys Acta.* 2011;1814(8):1030–1041. 10.1016/j.bbapap.2010.07.018 20656073

[19] Vogel KM Kozlowski PM Zgierski MZ: Determinants of the FeXO (X = C, N, O) vibrational frequencies in heme adducts from experiment and density functional theory. *J Am Chem Soc.* 1999;121(43):9915–9921 10.1021/ja990042r

[20] Li T Quillin ML Phillips GN Jr: Structural determinants of the stretching frequency of CO bound to myoglobin. *Biochemistry.* 1994;33(6):1433–1446. 10.1021/bi00172a021 8312263

[21] Ray GB Li XY Ibers JA: How far can proteins bend the FeCO unit? Distal polar and steric effects in heme proteins and models. *J Am Chem Soc.* 1994;116(1):162–176 10.1021/ja00080a019

[22] Spiro TG Ibrahim M Wasbotten IH: Chapter 4 - CO, NO, and O2 as Vibrational Probes of Heme Protein Active Sites. in *The Smallest Biomolecules: Diatomics and their Interactions with Heme Proteins*(Ghosh, A. ed.), Elsevier, Amsterdam.2008; pp95–123 10.1016/B978-044452839-1.50005-X

[23] Ewing GE: Infrared Spectra of Liquid and Solid Carbon Monoxide. *J Chem Phys.* 1962;37(10):2250 10.1063/1.1732994

[24] Franzen S Wallace-Williams SE Shreve AP: Heme charge-transfer band III is vibronically coupled to the Soret band. *J Am Chem Soc.* 2002;124(24):7146–7155. 10.1021/ja0172722 12059240

[25] Kriegl JM Nienhaus K Deng P: Ligand dynamics in a protein internal cavity. *Proc Natl Acad Sci U S A.* 2003;100(12):7069–7074. 10.1073/pnas.1231856100 12773621 PMC165831

[26] Park ES Andrews SS Hu RB: Vibrational stark spectroscopy in proteins: A probe and calibration for electrostatic fields. *J Phys Chem B.* 1999;103(45):9813–9817 10.1021/jp992329g

[27] Park ES Boxer SG: Origins of the sensitivity of molecular vibrations on electric fields: Carbonyl and Nitrosyl Stretches in Model Compounds and Proteins. *J Phys Chem.* 2002;106(22):5800–5806 10.1021/jp0203043

[28] Park ES Thomas MR Boxer SG: Vibrational Stark Spectroscopy of NO bound to Heme: Effects of Protein Electrostatic fields on the NO Stretch Frequency. *J Am Chem Soc.* 2000;122(49):12297–12303 10.1021/ja0014741

[29] Nienhaus K Olson JS Franzen S: The origin of stark splitting in the initial photoproduct state of MbCO. *J Am Chem Soc.* 2005;127(1):40–41. 10.1021/ja0466917 15631438

[30] Kushkuley B Stavrov SS: Theoretical study of the distal-side steric and electrostatic effects on the vibrational characteristics of the FeCO unit of the carbonylheme proteins and their models. *Biophys J.* 1996;70(3):1214–1229. 10.1016/S0006-3495(96)79680-7 8785279 PMC1225049

[31] Nutt DR Meuwly M: Theoretical investigation of infrared spectra and pocket dynamics of photodissociated carbonmonoxy myoglobin. *Biophys J.* 2003;85(6):3612–3623. 10.1016/S0006-3495(03)74779-1 14645054 PMC1303666

[32] Nienhaus K Deng P Kriegl JM: Structural dynamics of myoglobin: effect of internal cavities on ligand migration and binding. *Biochemistry.* 2003;42(32):9647–9658. 10.1021/bi034788k 12911306

[33] Nienhaus K Deng P Kriegl JM: Structural dynamics of myoglobin: spectroscopic and structural characterization of ligand docking sites in myoglobin mutant L29W. *Biochemistry.* 2003;42(32):9633–9646. 10.1021/bi034787s 12911305

[34] Lehle H Kriegl JM Nienhaus K: Probing electric fields in protein cavities by using the vibrational stark effect of carbon monoxide. *Biophys J.* 2005;88(3):1978–1990. 10.1529/biophysj.104.048140 15596507 PMC1305250

[35] Nienhaus K Maes EM Weichsel A: Structural dynamics controls nitric oxide affinity in nitrophorin 4. *J Biol Chem.* 2004;279(38):39401–39407. 10.1074/jbc.M406178200 15258143

[36] Lim M Jackson TA Anfinrud PA: Ultrafast rotation and trapping of carbon monoxide dissociated from myoglobin. *Nat Struct Biol.* 1997;4(3):209–214. 10.1038/nsb0397-209 9164462

[37] Bredenbeck J Helbing J Nienhaus K: Protein ligand migration mapped by nonequilibrium 2D-IR exchange spectroscopy. *Proc Natl Acad Sci U S A.* 2007;104(36):14243–14248. 10.1073/pnas.0607758104 17261808 PMC1964829

[38] Nienhaus K Knapp JE Palladino P: Ligand migration and binding in the dimeric hemoglobin of Scapharca inaequivalvis. *Biochemistry.* 2007;46(49):14018–14031. 10.1021/bi7016798 18001141 PMC2526229

[39] Nienhaus GU Heinzl J Huenges E: Protein Crystal Dynamics Studied by Time-resolved Analysis of X-ray Diffuse Scattering. *Nature.* 1989;338:665–666 10.1038/338665a0

[40] Frauenfelder H Nienhaus GU Johnson JB: Rate Processes in Proteins. *Ber Bunsenges Phys Chem.* 1991;95(3):272–278 10.1002/bbpc.19910950310

[41] Berendzen J Braunstein D: Temperature-derivative spectroscopy: a tool for protein dynamics. *Proc Natl Acad Sci U S A.* 1990;87(1):1–5. 10.1073/pnas.87.1.1 2296572 PMC53187

[42] Nienhaus GU Mourant JR Chu K: Ligand binding to heme proteins: the effect of light on ligand binding in myoglobin. *Biochemistry.* 1994;33(45):13413–13430. 10.1021/bi00249a030 7947750

[43] Mourant JR Braunstein DP Chu K: Ligand binding to heme proteins: II. Transitions in the heme pocket of myoglobin. *Biophys J.* 1993;65(4):1496–1507. 10.1016/S0006-3495(93)81218-9 8274643 PMC1225876

[44] Ghosh DK Wu C Pitters E: Characterization of the inducible nitric oxide synthase oxygenase domain identifies a 49 amino acid segment required for subunit dimerization and tetrahydrobiopterin interaction. *Biochemistry.* 1997;36(35):10609–10619. 10.1021/bi9702290 9271491

[45] Jung C Stuehr DJ Ghosh DK: FT-Infrared spectroscopic studies of the iron ligand CO stretch mode of iNOS oxygenase domain: effect of arginine and tetrahydrobiopterin. *Biochemistry.* 2000;39(33):10163–10171. 10.1021/bi0003792 10956005

[46] Li D Stuehr DJ Yeh SR: Heme distortion modulated by ligand-protein interactions in inducible nitric-oxide synthase. *J Biol Chem.* 2004;279(25):26489–26499. 10.1074/jbc.M400968200 15066989

[47] Crane BR Arvai AS Ghosh S: Structures of the N(omega)-hydroxy-L-arginine complex of inducible nitric oxide synthase oxygenase dimer with active and inactive pterins. *Biochemistry.* 2000;39(16):4608–4621. 10.1021/bi992409a 10769116

[48] Fukuto JM: Chemistry of N-hydroxy-L-arginine. *Methods Enzymol.* 1996;268:365–375. 10.1016/s0076-6879(96)68039-2 8782603

[49] Tantillo DJ Fukuto JM Hoffman BM: Theoretical studies on N-γ-hydroxy-L-arginine and derived radicals: Implications for the mechanism of nitric oxide synthase. *J Am Chem Soc.* 2000;122(3):536–537 10.1021/ja991876c

[50] Labby KJ Li HY Roman LJ: Methylated N-ω-Hydroxy-L-arginine Analogues as Mechanistic Probes for the Second Step of the Nitric Oxide Synthase-Catalyzed Reaction. *Biochemistry.* 2013;52(18):3062–3073. 10.1021/bi301571v 23586781 PMC3678535

[51] Lamb DC Nienhaus K Arcovito A: Structural dynamics of myoglobin: ligand migration among protein cavities studied by Fourier transform infrared/temperature derivative spectroscopy. *J Biol Chem.* 2002;277(14):11636–11644. 10.1074/jbc.M109892200 11792698

[52] Nienhaus K Lamb DC Deng P: The effect of ligand dynamics on heme electronic transition band III in myoglobin. *Biophys J.* 2002;82(2):1059–1067. 10.1016/S0006-3495(02)75465-9 11806945 PMC1301912

[53] Scott EE Gibson QH Olson JS: Mapping the pathways for O _2_ entry into and exit from myoglobin. *J Biol Chem.* 276(7):5177–5188. 10.1074/jbc.M008282200 11018046

[54] Nienhaus K Deng P Olson JS: Structural dynamics of myoglobin: ligand migration and binding in valine 68 mutants. *J Biol Chem.* 2003;278(43):42532–42544. 10.1074/jbc.M306888200 12907676

[55] Nienhaus K Zosel F Nienhaus GU: Ligand binding to heme proteins: a comparison of cytochrome c variants with globins. *J Phys Chem B.* 2012;116(40):12180–12188. 10.1021/jp306775n 22978708

[56] Nienhaus K Palladino P Nienhaus GU: Structural dynamics of myoglobin: FTIR-TDS study of NO migration and binding. *Biochemistry.* 2008;47(3):935–948. 10.1021/bi701935v 18161992

[57] Soldatova AV Ibrahim M Olson JS: New light on NO bonding in Fe(III) heme proteins from resonance Raman spectroscopy and DFT modeling. *J Am Chem Soc.* 132(13):4614–4625. 10.1021/ja906233m 20218710 PMC2853766

[58] Praneeth VK Paulat F Berto TC: Electronic structure of six-coordinate iron(III)-porphyrin NO adducts: the elusive iron(III)-NO(radical) state and its influence on the properties of these complexes. *J Am Chem Soc.* 2008;130(46):15288–15303. 10.1021/ja801860u 18942830

[59] Batabyal D Yeh SR: Human tryptophan dioxygenase: a comparison to indoleamine 2,3–dioxygenase. *J Am Chem Soc.* 2007;129(50):15690–15701. 10.1021/ja076186k 18027945

[60] Xu N Powell DR Cheng L: The first structurally characterized nitrosyl heme thiolate model complex. *Chem Commun (Camb).* 2006; (19):2030–2032. 10.1039/B602611G 16767265

[61] Obayashi E Tsukamoto K Adachi S: Unique binding of nitric oxide to ferric nitric oxide reductase from *Fusarium oxysporum* elucidated with infrared, resonance Raman, and X-ray absorption spectroscopies. *J Am Chem Soc.* 1997;119:7807–7816 10.1021/ja9637816

[62] Li H Igarashi J Jamal J: Structural studies of constitutive nitric oxide synthases with diatomic ligands bound. *J Biol Inorg Chem.* 2006;11(6):753–768. 10.1007/s00775-006-0123-8 16804678

[63] McQuarters AB Wirgau NE Lehnert N: Model complexes of key intermediates in fungal cytochrome P450 nitric oxide reductase (P450nor). *Curr Opin Chem Biol.* 2014;19:82–89. 10.1016/j.cbpa.2014.01.017 24658055

[64] Shimizu H Obayashi E Gomi Y: Proton delivery in NO reduction by fungal nitric-oxide reductase. Cryogenic crystallography, spectroscopy, and kinetics of ferric-NO complexes of wild-type and mutant enzymes. *J Biol Chem.* 2000;275(7):4816–4826. 10.1074/jbc.275.7.4816 10671516

[65] Pant K Crane BR: Nitrosyl-heme structures of *Bacillus subtilis* nitric oxide synthase have implications for understanding substrate oxidation. *Biochemistry.* 2006;45(8):2537–2544. 10.1021/bi0518848 16489746

[66] Goodrich LE Paulat F Praneeth VK: Electronic structure of heme-nitrosyls and its significance for nitric oxide reactivity, sensing, transport, and toxicity in biological systems. *Inorg Chem.* 2010;49(14):6293–6316. 10.1021/ic902304a 20666388

[67] Couture M Adak S Stuehr DJ: Regulation of the properties of the heme-NO complexes in nitric-oxide synthase by hydrogen bonding to the proximal cysteine. *J Biol Chem.* 2001;276(41):38280–38288. 10.1074/jbc.M105341200 11479310

[68] Rousseau DL Li D Hayden EY: Chapter 17 - Ligand-Protein Interactions in Mammalian Nitric Oxide Synthase. in *The Smallest Biomolecules: Diatomics and their Interactions with Heme Proteins*(Ghosh, A. ed.), Elsevier, Amsterdam.2008;465–497 10.1016/B978-044452839-1.50018-8

[69] Hu SZ Kincaid JR: Resonance Raman Spectra of the Nitric Oxide Adducts of Ferrous Cytochrome P450cam in the Presence of Various Substrates. *J Am Chem Soc.* 1991;113(26):9760–9766 10.1021/ja00026a008

[70] Praneeth VK Haupt E Lehnert N: Thiolate coordination to Fe(II)-porphyrin NO centers. *J Inorg Biochem.* 2005;99(4):940–948. 10.1016/j.jinorgbio.2005.02.007 15811511

[71] Li D Hayden EY Panda K: Regulation of the monomer-dimer equilibrium in inducible nitric-oxide synthase by nitric oxide. *J Biol Chem.* 2006;281(12):8197–8204. 10.1074/jbc.M507328200 16421101

[72] Abu-Soud HM Wu C Ghosh DK: Stopped-flow analysis of CO and NO binding to inducible nitric oxide synthase. *Biochemistry.* 1998;37(11):3777–3786. 10.1021/bi972398q 9521697

[73] Wang J Rousseau DL Abu-Soud HM: Heme coordination of NO in NO synthase. *Proc Natl Acad Sci U S A.* 1994;91(22):10512–10516. 10.1073/pnas.91.22.10512 7524095 PMC45051

[74] Ibrahim M Xu CL Spiro TG: Differential sensing of protein influences by NO and CO vibrations in heme adducts. *J Am Chem Soc.* 2006;128(51):16834–16845. 10.1021/ja064859d 17177434 PMC2530899

[75] Coyle CM Vogel KM Rush TS 3rd: FeNO structure in distal pocket mutants of myoglobin based on resonance Raman spectroscopy. *Biochemistry.* 2003;42(17):4896–4903. 10.1021/bi026395b 12718530

[76] Chiavarino B Crestoni ME Fornarini S: Direct probe of NO vibration in the naked ferric heme nitrosyl complex. *Chemphyschem.* 2008;9(6):826–828. 10.1002/cphc.200800086 18383061

[77] Lanucara F Chiavarino B Crestoni ME: Naked five-coordinate Fe(III)(NO) porphyrin complexes: vibrational and reactivity features. *Inorg Chem.* 2011;50(10):4445–4452. 10.1021/ic200073v 21476565

[78] Dey A Okamura TA Ueyama N: Sulfur K-edge XAS and DFT calculations on P450 model complexes: effects of hydrogen bonding on electronic structure and redox potentials. *J Am Chem Soc.* 2005;127(34):12046–12053. 10.1021/ja0519031 16117545 PMC2880190

[79] Decatur SM Franzen S DePillis GD: Trans effects in nitric oxide binding to myoglobin cavity mutant H93G. *Biochemistry.* 1996;35(15):4939–4944. 10.1021/bi951661p 8664286

[80] Fernandez ML Marti MA Crespo A: Proximal effects in the modulation of nitric oxide synthase reactivity: a QM-MM study. *J Biol Inorg Chem.* 2005;10(6):595–604. 10.1007/s00775-005-0004-6 16133202

[81] Linder DP Rodgers KR Banister J: Five-coordinate Fe(III)NO and Fe(II)CO porphyrinates: where are the electrons and why does it matter? *J Am Chem Soc.* 2004;126(43):14136–14148. 10.1021/ja046942b 15506779 PMC1525220

[82] Huang L Abu-Soud HM Hille R: Nitric oxide-generated P420 nitric oxide synthase: characterization and roles for tetrahydrobiopterin and substrate in protecting against or reversing the P420 conversion. *Biochemistry.* 1999;38(6):1912–1920. 10.1021/bi981954t 10026272

[83] Horn M Nienhaus K Nienhaus GU: Fourier transform infrared photolysis difference spectra of CO- and NO-ligated inducible nitric oxide synthase. *F1000Research.* 2014 Data Source 10.12688/f1000research.5836.1 PMC4304226 25653844

