# Supplementary material for: Fourier transform infrared spectroscopy study of ligand photodissociation and migration in inducible nitric oxide synthase
Source: F1000Res. 2014 Dec 12;3:290. Originally published 2014 Nov 28. [Version 2] doi: 10.12688/f1000research.5836.2 (PMC4304226; doi:10.12688/f1000research.5836.2)
Supplement: Peer review history

# Peer review: Referee response for version 1

**DOI:** 10.5256/f1000research.6238.r6849

**Published:** 2014-12-05

**Authors:** Marius Schmidt

**Affiliations:** 1 Department of Physics, University of Wisconsin-Milwaukee, Milwaukee, WI, USA

This is a very interesting investigation on protein ligand interaction in iNOS. It is well written and informative. It should be indexed, and requires only minor revisions.

- p.6 text: ”In substrate free iNOSoxy-CO, recombination is already maximal at 4 K and extends to ~70 K.”
  What does this mean? What is a maximal recombination?
  Suggestion: “there is already substantial recombination at 4 K and the process extends to 70 K.”

 

- p. 6 text: “A population transfer between photoproduct states due to CO rotation 32,51,52 is apparent from the mirror-imaged dashed and solid contours at 2131 and 2144 cm-1 at 12 K”.
  Just write a sentence or two why that is so. The non-expert reader should not read the literature for this.

 

- Figure 3, caption: there is a mix-up with panel numbers.

 

- The case for a 5C and the 4C (naked) heme is well made. This is a very interesting result and merits closer investigation by X-ray structure determination.

I have read this submission. I believe that I have an appropriate level of expertise to confirm that it is of an acceptable scientific standard.

# Peer review: Referee response for version 1

**DOI:** 10.5256/f1000research.6238.r6853

**Published:** 2014-12-05

**Authors:** Pál Ormos

**Affiliations:** 1 Institute of Biophysics, Biological Research Centre of the Hungarian Academy of Sciences, Szeged, Hungary

It is investigated how ligands NO and CO dissociate and bind to inducible nitric oxide synthase. The applied method is FTIR difference spectroscopy, in particular low temperature temperature derivative spectroscopy, most appropriate to elucidate details of the process. The binding route has been clarified and compared to related ligand binding heme proteins. The results are important in characterizing the enzyme. As I understand, the task of the referee is primarily to judge the soundness, the technical quality of the work. There is absolutely no problem in this respect: the method is appropriate, executed perfectly, the conclusions are well supported. I suggest indexing without any modification.

I have read this submission. I believe that I have an appropriate level of expertise to confirm that it is of an acceptable scientific standard.
